# Interventions to Prevent Post-Discharge Mortality among Children in Sub-Saharan Africa: A Systematic Review

**DOI:** 10.4269/ajtmh.25-0567

**Published:** 2026-02-17

**Authors:** Kaitlin Cole, Ramya Ginjupalli, Karim P. Manji, Rodrick Kisenge, Adrianna Westbrook, Quique Bassat, Rosauro Varo, Lola Madrid, Inacio Mandomando, Claudia R. Morris, Hannah Rogers, Nega Assefa, Richard Omore, Victor Akelo, Kitiezo Aggrey Igunza, Christopher P. Duggan, Chris A. Rees

**Affiliations:** ^1^ Emory University School of Medicine, Atlanta, Georgia;; ^2^ Department of Pediatrics and Child Health, Muhimbili University of Health and Allied Sciences, Dar es Salaam, Tanzania;; ^3^ Pediatric Biostatistics Core, Department of Pediatrics, Emory University, Atlanta, Georgia;; ^4^ ISGlobal, Barcelona, Spain;; ^5^ Centro de Investigação em Saúde de Manhiça, Maputo, Mozambique;; ^6^ ICREA, Barcelona, Spain;; ^7^ Institut Clínic de Medicina I Dermatologia, Hospital Clínic de Barcelona, Barcelona, Spain;; ^8^ Facultat de Medicina i Ciències de la Salut, Universitat de Barcelona, Barcelona, Spain;; ^9^ Pediatrics Department, Hospital Sant Joan de Déu, Universitat de Barcelona, Barcelona, Spain;; ^10^ CIBER de Epidemiología y Salud Pública, Instituto de Salud Carlos III, Madrid, Spain;; ^11^ London School of Hygiene and Tropical Medicine, London, United Kingdom;; ^12^ College of Health and Medical Sciences, Haramaya University, Harar, Ethiopia;; ^13^ Instituto Nacional de Saúde, Ministério de Saúde, Maputo, Mozambique;; ^14^ Division of Pediatric Emergency Medicine, Emory University School of Medicine, Atlanta, Georgia;; ^15^ Children’s Healthcare of Atlanta, Atlanta, Georgia;; ^16^ Woodruff Health Sciences Center Library, Emory University, Atlanta, Georgia;; ^17^ Kenya Medical Research Institute-Center for Global Health Research, Kisumu, Kenya;; ^18^ Liverpool School of Tropical Medicine, Liverpool, United Kingdom;; ^19^ Departments of Nutrition and Global Health and Population, Harvard T.H. Chan School of Public Health, Boston, Massachusetts;; ^20^ Center for Nutrition, Division of Gastroenterology, Hepatology, and Nutrition, Boston Children’s Hospital, Boston, Massachusetts

## Abstract

Post-discharge mortality (PDM), defined as deaths that occur in the weeks and months after hospital discharge, remains a critical, yet under-recognized, contributor to high childhood mortality rates in sub-Saharan Africa. However, a comprehensive understanding of effective interventions to prevent PDM is lacking. The aim for the present study was to evaluate the efficacy of published interventions to prevent PDM among neonates and children aged 0–18 years in sub-Saharan Africa. A systematic review was conducted to assess the efficacy of interventions for preventing PDM. The CABI Global Health, Cochrane Reviews, Cochrane Trials, ProQuest Dissertations and Theses, Embase, PubMed, and Web of Science databases were searched without language restriction. Publications that involved interventions for preventing PDM, included children, and were conducted in sub-Saharan Africa were included in the present study. Of 4,893 publications screened, 17 were included, with 12,938 participants in total (10.6% experienced PDM). The most common interventions included supplemental feeding programs, kangaroo mother care, antibiotic use, and micronutrient supplementation. Effectiveness varied within and between intervention types. Only two interventions resulted in statistically significant reductions in PDM: vitamin A supplementation for children with pneumonia (hazard ratio: 0.51; 95% CI: 0.29–0.90; low quality of evidence) and linkage to services for children with sickle cell disease (adjusted hazard ratio: 0.26; 95% CI: 0.08–0.83; low quality of evidence). No single intervention type provided consistent benefits across studies. Most interventions targeted children with specific diagnoses; however, some strategies addressed social determinants of health. Future research must prioritize cost-effective, scalable strategies across diverse sub-Saharan African settings to accelerate the prevention of PDM among children.

## INTRODUCTION

Globally, childhood mortality rates have declined by more than 50% between the 1990s and the 2020s.^1^ Nonetheless, reductions in childhood mortality remain geographically unequal, as sub-Saharan African countries contribute to 60% of all childhood deaths globally. Childhood mortality rates among children in sub-Saharan Africa are 17 times those observed in high-income countries.^2^ The large reductions in childhood mortality from the 1990s to the 2020s have been due, in part, to advances in sanitation and hygiene, widespread vaccinations, and clinical care.^3^ However, there are growing concerns that reductions in childhood mortality rates are slowing.^4^

Deaths that occur in the weeks and months after hospital discharge, known as post-discharge mortality (PDM), contribute substantially to high childhood mortality rates in sub-Saharan Africa, yet have not historically been targeted in policy and practice.^5^ Evidence from recent studies indicates that PDM rates among children of all ages in sub-Saharan Africa are as high as 3–13%, with several studies suggesting that PDM rates may outpace those observed during hospitalization.^6^^,^^7^ Despite its contribution to persistently high rates of childhood mortality in sub-Saharan Africa, studies on PDM have lagged behind those on inpatient mortality, resulting in a paucity of implemented approaches to prevent PDM.^8^ Previous systematic reviews and meta-analyses have focused on the prevalence of PDM and its associated risk factors, including malnutrition, HIV status, unplanned discharges, young age, and leaving the hospital against medical advice (among others).^6^^,^^7^^,^^9^ However, a comprehensive understanding of feasible and effective interventions for preventing PDM among children in sub-Saharan Africa is lacking. The unique combination of high infectious disease prevalence, malnutrition, limited healthcare infrastructure, and challenges in post-discharge follow-up in sub-Saharan Africa necessitates a targeted evaluation of post-discharge interventions for this setting.^6^

To better inform potentially effective interventions to prevent PDM, the aim for the present study was to evaluate the efficacy of published interventions for preventing PDM among children in sub-Saharan Africa. Understanding effective interventions for preventing PDM among children may better inform areas for further investigation and implementation in sub-Saharan Africa.

## MATERIALS AND METHODS

### Study design.

A systematic review was conducted in accordance with the Cochrane methodology and the Preferred Reporting Items for Systematic Reviews and Meta-Analyses framework to identify publications reporting on the efficacy of interventions for preventing PDM among children in sub-Saharan Africa.^10^ The study protocol was registered prospectively in PROSPERO (CRD42024577326).^11^

### Search strategy.

Publications that reported on interventions for preventing PDM among neonates and children (defined as individuals aged 0–18 years) in sub-Saharan Africa were included. Sub-Saharan African countries were identified according to the World Bank’s classification.^12^ The search included the CABI Global Health, Cochrane Reviews, Cochrane Trials, ProQuest Dissertations and Theses, Embase (Elsevier), PubMed, and Web of Science databases, retrieving all publications from each database’s inception to the date the search was conducted (i.e., July 30, 2024). No restrictions were placed on article type, length of follow-up after discharge, date of publication, or language. The search strategy (Appendix) was developed in collaboration with a librarian skilled in systematic reviews.

### Inclusion and exclusion criteria.

Publications were included if they 1) involved interventions for preventing PDM due to any cause among children, 2) were published in a peer-reviewed journal (verified via journal title and author guidelines), 3) included children aged 0–18 years, and 4) were conducted exclusively or partially in at least one sub-Saharan African country. Publications were excluded if they included adults (defined as individuals aged >18 years) and did not distinguish outcomes between children and adults, or if they involved interventions for preventing PDM but occurred exclusively outside sub-Saharan Africa. Because no pre-determined categories of interventions were defined, all publications that involved interventions initiated at hospital discharge, initiated during hospitalization and continued post-discharge, or initiated after discharge with the outcome of PDM over any reported timeframe were included. Once publications were identified, interventions were grouped by type (e.g., antibiotics administered after discharge, micronutrient supplementation after discharge, etc.). Randomized controlled trials, non-randomized trials, and observational cohort studies that involved interventions for reducing PDM were included. Case reports, interventions without a comparison group, and studies lacking a clear intervention component were ultimately excluded. Studies of malaria chemoprophylaxis were excluded, given that this intervention had been reviewed in a recent systematic review and meta-analysis, and no additional published trials beyond those included in that review were found.^13^^–^^16^

### Selection process.

After the initial search, records were downloaded using EndNote and imported into Covidence. During the screening phase, each title and abstract was independently evaluated by two of three authors (K.C., R.G., and C.A.R.) to assess eligibility. Titles and abstracts deemed ineligible for inclusion by two authors were removed from consideration. In cases of disagreement between two reviewers, a third reviewer independently assessed the title and abstract for potential inclusion without knowledge of the previous reviewers’ recommendations to make a final determination regarding inclusion or exclusion. Titles and abstracts that passed the screening phase underwent a full-text review for potential inclusion using the same two-person review process. To ensure that all available published evidence was included, the references cited in each included publication were reviewed to identify any additional publications that may have met the inclusion criteria but were missed during the initial search.

### Data extraction.

Two authors (R.G. and K.C.) independently reviewed and extracted data from full-text publications into a structured data extraction tool using a Microsoft Excel® file (Microsoft Corp., Redmond, WA). All entered data were reviewed by a third reviewer (C.A.R.) to assess data quality and consistency. Any discrepancies in the extracted data were resolved through discussion and assessment of the original publication by a third reviewer (C.A.R.).

For each publication that met the inclusion criteria, the following characteristics were extracted: study country and site, age groups included, study design, sample size, number of participants in the intervention and control groups (when included) who had the outcome of PDM, number lost to follow up, hospital diagnoses of included cases, interventions, follow-up time period, measured outcome, and measure of efficacy as reported.

### Risk of bias assessment.

To assess bias in studies on interventions for preventing PDM, the Risk of Bias 2 (ROB-2) tool (Cochrane Collaboration, London, United Kingdom) was employed, which is used to assess risk of bias in randomized controlled trials.^17^ This assessment was completed independently by two authors (K.C. and R.G.) and reviewed for disagreement by an arbiter (C.A.R.). Disagreements regarding ROB-2 determinations were discussed until the three reviewers reached consensus.

### Assessment of certainty of evidence.

For publications that met the inclusion criteria, the certainty of evidence was assessed using the Grades of Recommendation, Assessment, Development, and Evaluation (GRADE) Working Group system.^18^ The GRADE handbook and previous studies of randomized controlled trials for interventions were used as guidelines for completing the GRADE assessment.^19^

## STATISTICAL ANALYSES

Descriptive statistics were used to evaluate study characteristics. Effect sizes (i.e., risk or hazard ratios) were calculated using data reported in each study. For studies within the same intervention category, heterogeneity was assessed by calculating I^2^. Additionally, random-effects models were used to account for heterogeneity due to study design differences, such as in age, location, or variations in intervention. Two trials with a 2 × 2 factorial design and two distinct interventions were found. These were treated as separate interventions because the interaction between the two interventions in those assigned to receive both (i.e., antibiotics and micronutrients) was not reported separately in the included publication. For one of these trials,^20^ the results of the second intervention (i.e., Bacillus Calmette-Guérin [BCG] vaccination) were noted to have been reported elsewhere; after identifying this, the separately reported intervention was found and included in the list of studies. Because the identified interventions differed in the specific types and follow-up periods used to assess PDM outcomes, a meta-analysis of identified studies was not conducted. Instead, all results were presented as reported in the primary publication. All analyses were conducted using R V.4.0.3 (R Foundation for Statistical Computing, Vienna, Austria).

## RESULTS

**Figure 1.**
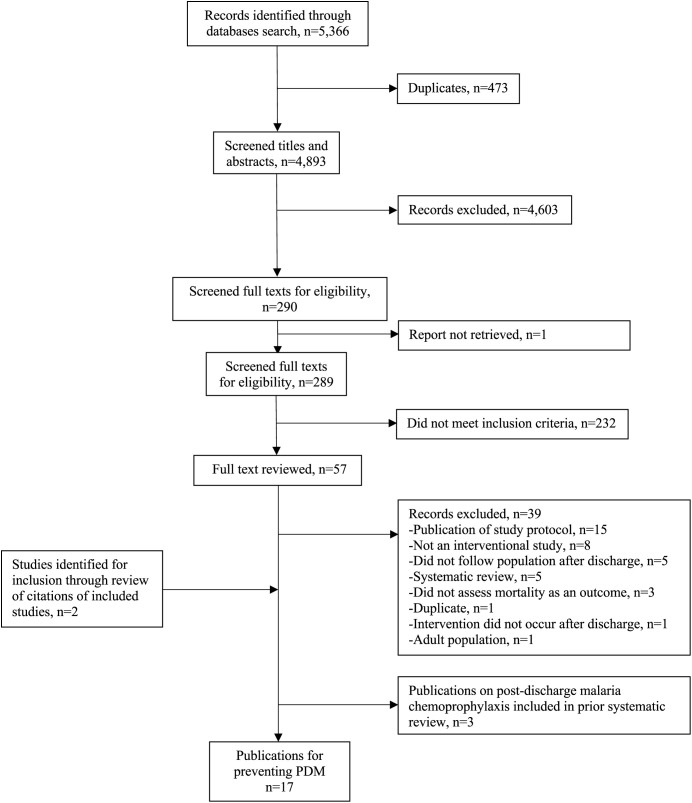
Preferred Reporting Items for Systematic Reviews and Meta-Analyses diagram of included publications on interventions for preventing post-discharge mortality among children in sub-Saharan Africa.

The literature search identified 4,893 unique publications. After the titles and abstracts were screened, 289 full-text publications were assessed for eligibility, of which 15 met the inclusion criteria and involved interventions for preventing PDM among children in sub-Saharan Africa (Figure 1). Two studies were later included after a review of the citations of the included publications. Therefore, a total of 17 publications were included.

### Characteristics of included publications.

In the included publications, the efficacy of 18 distinct interventions for preventing PDM was described (Table 1). The duration of post-discharge follow-up ranged from 28 days to >24 months. The studied interventions included supplemental feeding after hospital discharge (*n* = 4 studies), kangaroo mother care (KMC) initiated during hospital admission and continued after discharge (*n* = 3 studies), antibiotic use after hospital discharge (*n* = 3 studies), micronutrient supplementation after hospital discharge (*n* = 4 studies), BCG vaccination at hospital discharge (*n* = 1), linkage to care with social workers (*n* = 1), antiretroviral therapy initiation during admission and continuation after discharge (*n* = 1), and referrals for scheduled visits and supplies, including soaps and oral rehydration salts (*n* = 1).

**Table 1:** Description of included studies on interventions for preventing post-discharge mortality

Reference	Study Countries	Age Groups Included	Study Design	Healthcare Facility	Sample Size, *n*	Number Who Had Outcome, *n* (%)	Number Lost to Follow-up, *n* (%)	Follow-up Period	Intervention	Measured Outcome
Supplemental feeding										
Kiguli S, et al. *EClinicalMedicine*. 2024.^21^	Uganda, Kenya	6 months to 12 years	Open-label phase 2 randomized controlled trial	Mbale, Soroti, Jinja, Maska Regional Referral Hospitals, Uganda and Kilifi County Hospital	846	33 (4.0%)	12 (1.4%)	6 months	Usual diet + RUTF for children hospitalized with severe pneumonia	90-day mortality
Rollins NC, et al. *Acta Paediatr.* 2007.^22^	South Africa	6–36 months	Randomized non-blinded study	King Edward VIII Hospital	169	43 (25.4%)	22 (13.0%)	26 weeks	Enhanced diet for HIV-infected children with prolonged diarrhea	26-week mortality
Walsh K, et al. *Br J Nutr*. 2024.^23^	Uganda	6 months to 5 years	Open-label phase II trial	Mbale and Soroti Regional Referral Hospitals	160	23 (14.4%)	16 (10.0%)	90 days	Lactose-free, chickpea-enriched legume paste feed for severe malnutrition	90-day mortality
Kerac M, et al. *Lancet.* 2009.^24^	Malawi	5–168 months	Double blind randomized placebo-controlled efficacy trial	Queen Elizabeth Central Hospital	795	227 (28.6%)	10 (1.3%)	10 weeks	Synbiotic fortified RUTF	10-week mortality
KMC										
Brotherton H, et al. *EclinicalMedicine*. 2021.^25^	The Gambia	Neonates	Nonblinded pragmatic randomized controlled trial	Edward Francis Small Teaching Hospital	279	63 (22.5%)	2 (0.7%)	28 days	KMC initiated within 24 hours of birth	28-day mortality
Kambarami RA, et al. *Ann Trop Paediatr.* 2003.^26^	Zimbabwe	Neonates	Prospective descriptive study	Harare Hospital	297	79 (26.6%)	77 (25.9%)	12 months	KMC for preterm infants weighing 500–1,800 g	12-month mortality
Nagai S, et al. *Acta Paediatr*. 2011.^27^	Madagascar	Neonates	Randomized controlled trial with long-term follow-up	University Hospital of Mahajanga	72	4 (5.6%)	1 (1.4%)	1 year	KMC initiated within 24 hours of birth	6- to 12-month mortality
*Antibiotics*										
Berkley JA, et al. *Lancet Glob Health*. 2016.^28^	Kenya	60 days to 59 months	Multicentre, double-blind, randomized, placebo-controlled study	Kilifi County Hospital, Coast General Hospital Mombasa, Malindi sub-County Hospital, Mbagathi Hospital Nairobi	1,778	257 (14.5%)	56 (3.1%)	12 months	Cotrimoxazole prophylaxis for children with severe acute malnutrition	12-month mortality
Pavlinac PB, et al. *Lancet Glob Health*. 2021.^29^	Kenya	1–59 months	Double blind placebo controlled randomized controlled trial	Kisii Teaching and Referral Hospital, Homa Bay Teaching and Referral Hospital, St Paul Mission Hospital, Kendu Adventist Mission Hospital	1,400	34 (2.4%)	2 (0.14%)	6 months	Azithromycin 5-day course at hospital discharge	6-month mortality
Maitland K, et al. *Lancet Glob Health*. 2019.^30^	Uganda, Malawi	2 months to 12 years	Two-stratum open-label multicenter factorial randomized tract trial	Mulago Hospital, Mbale Regional Referral Hospital, Soroti Regional Referral Hospital, University of Malawi	3,983	335 (8.4%)	164 (4%)	180 days	Cotrimoxazole prophylaxis for children with severe anemia	180-day mortality
Micronutrient supplementation										
Benn CS, et al. *BMJ*. 2010.^16^	Guinea-Bissau	Neonates	Randomized placebo-controlled two-by-two factorial trial	Guinea-Bissau National Hospital	1,717	161 (9.4%)	145 (8.4%)	12 months	Vitamin A supplementation	12-month mortality
Fawzi, et al. *Pediatr Infect Dis J.* 1999.^31^	Tanzania	6 months to 5 years	Randomized double blind placebo- controlled trial	Muhimbili Medical Center	687	52 (7.6%)	76 (11.1%)	Mean of 24.4 months	Vitamin A at discharge, 4 months, and 8 months for children admitted for pneumonia	Mortality after mean follow-up of 24.4 months
Maitland K, et al. *Lancet Glob Health.* 2019.^30^	Uganda, Malawi	2 months to 12 years	Two-stratum open-label multicentre factorial randomized tract trial	Mulago Hospital, Mbale Regional Referral Hospital, Soroti Regional Referral Hospital, University of Malawi	3,983	335 (8.4%)	164 (4.1%)	180 days	Multivitamin multimineral supplement	180-day mortality
Makonnen B, et al. *J Trop Pediatr*. 2003.^32^	Lesotho	6–60 months	Randomized controlled trial	Queen Elizabeth II Hospital	254	2 (0.8%)	7 (2.8%)	3 months	10 mg elemental zinc supplementation	3-month mortality
Other interventions										
Aaby, et al. *J Infectious Diseases.* 2011.^33^	Guinea-Bissau	Neonates	Randomized placebo-controlled two-by-two factorial trial	Guinea-Bissau National Hospital	2,320	229 (9.9%)	Not reported	12 months	BCG vaccination	12-month mortality
Hau DK, et al. *J Pediatr.* 2021.^34^	Tanzania	6–144 months	Pilot study	Bugando Medical Center	116	15 (12.9%)	0 (0.0%)	1 year	Linkage to care with social workers	12-month mortality
Njuguna, et al. *Topics in antiviral medicine.* 2016.^35^	Kenya	0–12 years	Unmasked RCT	Kenyatta National Hospital, Mbagathi District Hospital, Jarmogi Oginga Hospital, Kisumu County Hospital	183	39 (21.3%)	14 (7.6%)	6 months	anti-retroviral therapy within 48 hours	6-month mortality
Wiens MO, et al. *Glob Health Sci Pract*. 2016.^36^	Uganda	6 months to 5 years	Proof of concept	Mbarara Regional Referral Hospital, Holy Innocents Children’s Hospital	202	5 (2.5%)	2 (1.0%)	60 days	1) Referrals for scheduled follow-up visits, 2) discharge counseling, and 3) simple prevention items, including soap and oral rehydration salts	60-day mortality

BCG = Bacillus Calmette-Guérin; KMC = kangaroo mother care; RUTF = ready-to-use therapeutic feeds.

A total of 15,258 participants were enrolled across the included studies, of whom 10.5% (*n* = 1,601) experienced PDM during the studies’ follow-up periods (Table 1). The included publications involved studies conducted in Uganda (*n* = 4), Kenya (*n* = 4), Malawi (*n* = 2), Tanzania (*n* = 2), the Gambia (*n* = 1), Guinea-Bissau (*n* = 2), Lesotho (*n* = 1), Madagascar (*n* = 1), South Africa (*n* = 1), and Zimbabwe (*n* = 1). All but two of the included publications revealed results from studies conducted in a single country. Participants’ ages ranged from 0 days to 14 years.

### Risk of bias.

Of the 18 reported interventions, eight had a low overall risk of bias, and seven were deemed to have some overall risk of bias (Supplemental Table 1). The remaining three interventions were not randomized controlled trials and, consequently, were not evaluated using the RoB-2 tool.

### Supplemental feeding programs.

**Figure 2.**
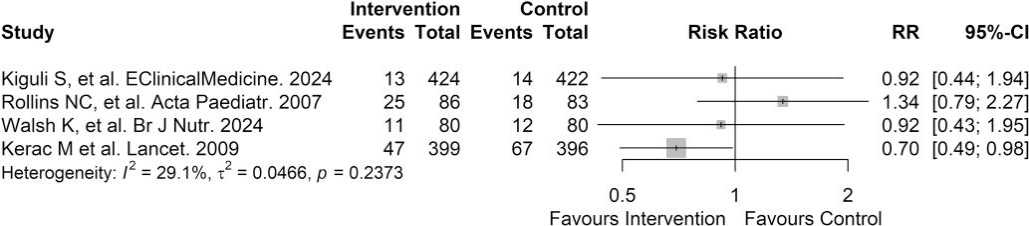
Forest plot of supplemental feeding interventions for preventing post-discharge mortality in sub-Saharan Africa.

Four studies, with a total of 1,970 participants, involved interventions for preventing PDM through supplemental feeding (Figure 2). One of these studies was a multicenter randomized controlled trial conducted in Uganda and Kenya that included 846 participants aged 6 months to 12 years admitted with severe pneumonia.^21^ The impact of supplementing the patient’s usual diet with ready-to-use therapeutic food after hospital discharge on 90-day mortality was assessed in the study (adjusted hazard ratio: 1.00; 95% CI: 0.46–2.15; moderate quality of evidence; Supplemental Tables 2 and 3). A single-center randomized controlled trial conducted in South Africa included 169 HIV-positive participants aged 6 to 36 months who had prolonged diarrheal disease and was used to assess the impact of supplementing a patient’s standard diet with powdered protein on weight gain as a primary outcome and 26-week mortality as a secondary outcome (22% mortality for the control group versus 29% mortality for the intervention group [significance not reported]; low quality of evidence; Supplemental Tables 2 and 3).^22^ A randomized controlled trial conducted at two sites in Uganda enrolled 160 participants aged 6 months to 5 years admitted with severe malnutrition to assess the impact of a lactose-free, chickpea-enriched legume food paste on 90-day mortality.^23^ The intervention yielded a hazard ratio of 0.91 (95% CI: 0.40–2.07; very low quality of evidence; Supplemental Tables 2 and 3). A single-center randomized controlled trial conducted in Malawi included 795 participants aged 5–168 months admitted with severe acute malnutrition and was used to assess the impact of Synbiotic fortified ready-to-use therapeutic food on 10-week mortality.^24^ The intervention yielded a risk ratio of 0.71 (95% CI: 0.51–1.00; moderate quality of evidence; Supplemental Tables 2 and 3).

### Kangaroo mother care.

Three studies, with a total of 648 participants, assessed KMC as an intervention for preventing PDM. A single-center randomized controlled trial conducted in the Gambia included 279 neonates and was used to assess the impact of KMC initiated within 24 hours of birth and continued after discharge on 28-day mortality (risk ratio: 0.84, 95% CI: 0.55–1.29; very low quality of evidence; Supplemental Tables 2 and 3).^25^ A prospective descriptive study in Zimbabwe included 297 pre-term neonates with birthweights of 500–1,800 grams and assessed the impact of KMC on 12-month mortality (26.6% mortality rate with no control group; very low quality of evidence; Supplemental Tables 2 and 3).^26^ A single-center randomized controlled trial conducted in Madagascar included 72 neonates and was used to assess the impact of KMC within 24 hours of birth on 6- to 12-month mortality and yielded a risk ratio of 1.00 (95% CI: 0.15–6.72; very low quality of evidence; Supplemental Tables 2 and 3).^27^

### Antibiotics.

**Figure 3.**
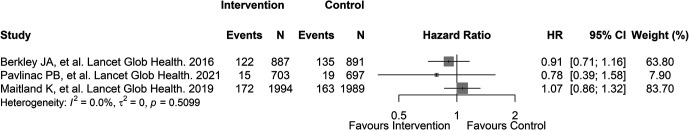
Forest plot of antibiotic usage after hospital discharge to prevent post-discharge mortality in sub-Saharan Africa.

Three studies with a total of 7,161 participants involved interventions for preventing PDM through antibiotic use after hospital discharge (Figure 3). A randomized controlled trial conducted at four sites in Kenya included 1,778 participants aged 60 days to 59 months admitted with severe acute malnutrition.^28^ The impact of once-daily oral co-trimoxazole prophylaxis administered for 6 months on 12-month mortality was assessed in the study, yielding a hazard ratio of 0.90 (95% CI: 0.71–1.16; moderate quality of evidence; Supplemental Tables 2 and 3). Another randomized controlled trial conducted at four hospitals in Kenya included 1,400 participants aged 1–59 months admitted for any reason other than trauma, poisoning, or congenital anomalies.^29^ The impact of a 5-day course of azithromycin at hospital discharge on 6-month PDM was assessed in the study (hazard ratio: 0.79; 95% CI: 0.39–1.58; very low quality of evidence; Supplemental Tables 2 and 3). A randomized controlled trial conducted at three sites in Uganda and one site in Malawi included 3,983 participants aged 2 months to 12 years admitted for severe anemia (i.e., hemoglobin levels <6 g/dL).^30^ The impact of a 90-day course of once-daily co-trimoxazole prophylaxis on 180-day PDM was assessed in the study, yielding an adjusted hazard ratio of 1.07 (95% CI: 0.86–1.32; low quality of evidence; Supplemental Tables 2 and 3).

### Micronutrient supplementation.

**Figure 4.**
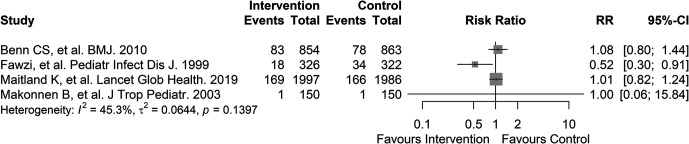
Forest plot of micronutrient supplementation after discharge to prevent post-discharge mortality in sub-Saharan Africa.

Four studies involving a total of 6,641 participants were conducted to assess micronutrient supplementation as an intervention for preventing PDM (Figure 4). A single-center randomized controlled trial conducted in Guinea-Bissau included 1,717 low-birth weight neonates (i.e., <2,500 grams).^16^ The impact of vitamin A supplementation on 12-month mortality was assessed in the study, yielding an adjusted risk ratio of 1.08 (95% CI: 0.79–1.47; very low quality of evidence; Supplemental Tables 2 and 3). A single-center randomized controlled trial conducted in Tanzania included 687 participants aged 6 months to 5 years admitted with pneumonia to assess the impact of vitamin A supplementation on PDM at discharge, 4 months after discharge, and 8 months after discharge, with a mean follow-up period of 24.4 months.^31^ The intervention yielded a hazard ratio of 0.51 (95% CI: 0.29–0.90; low quality of evidence; Supplemental Tables 2 and 3). A multicenter randomized controlled trial conducted at three sites in Uganda and one site in Malawi included 3,983 participants aged 2 months to 12 years admitted for severe anemia (i.e., hemoglobin levels <6 g/dL).^30^ The impact of multivitamin mineral supplementation on 180-day PDM compared with iron and folate supplementation was assessed in the study, yielding a hazard ratio of 0.97 (95% CI: 0.79–1.21; low quality of evidence; Supplemental Tables 2 and 3). A single-center randomized controlled trial conducted in Lesotho included 254 participants aged 6–60 months admitted with malnutrition to assess the impact of elemental zinc supplementation at a dosage of 10 mg on several outcomes, including the secondary outcome of 3-month PDM (0.9% mortality for control versus 0.7% mortality for intervention; very low quality of evidence; Supplemental Tables 2 and 3).^32^

### Other interventions.

A single-center randomized controlled trial conducted in Guinea-Bissau included 2,320 neonates with birthweights of <2,500 grams.^33^ The impact of BCG vaccination at discharge on 12-month mortality was assessed in the study, yielding a risk ratio of 0.83 (95% CI: 0.63–1.08; very low quality of evidence; Supplemental Tables 2 and 3). A single-center pilot study conducted in Tanzania included 116 pediatric participants with sickle cell disease.^34^ The impact of an intervention focused on linkage to clinical care with social workers after hospital discharge on 12-month PDM was assessed in the study (adjusted hazard ratio: 0.26; 95% CI: 0.08–0.83; very low quality of evidence; Supplemental Tables 2 and 3). A multicenter randomized controlled trial conducted in Kenya included 183 participants aged 0–12 years admitted with HIV to assess the impact of antiretroviral therapy initiation within 48 hours of admission and continuation after discharge on 6-month PDM.^35^ The intervention yielded an adjusted hazard ratio of 1.30 (95% CI: 0.69–2.45; very low quality of evidence; Supplemental Tables 2 and 3). A proof-of-concept study conducted at two sites in Uganda included 202 participants aged 6 months to 5 years who were admitted with suspected or proven infection to assess the impact of referrals for scheduled follow-up visits, discharge counseling, and prevention items, including soap and oral rehydration salts, on 60-day PDM.^36^ The intervention yielded an odds ratio of 0.75 (95% CI: 0.29–1.92; very low quality of evidence; Supplemental Tables 2 and 3).

## DISCUSSION

A total of 18 interventions for preventing PDM among children aged 0 to 14 years assessed in 10 sub-Saharan African countries were identified in the present systematic review. The most studied interventions for preventing PDM included supplemental feeding programs, KMC, antibiotic usage, and micronutrient supplementation. Other interventions included antiretroviral treatment, BCG vaccination, access to services, and post-discharge follow-up. Interventions, even within the same category, exhibited mixed effectiveness in preventing PDM among children, and none of the identified interventions were consistent across studies in terms of their intervention or follow-up periods.

The impact of supplemental feeding programs on preventing PDM among children was assessed in three studies. These programs have the potential to prevent PDM because undernutrition has been repeatedly identified as a risk factor for PDM, and it contributes to more than 40% of deaths in children under 5 years of age in sub-Saharan Africa.^37^^–^^40^ Kangaroo mother care was also commonly studied as an intervention for preventing PDM among neonates and young infants, likely because this low-resource, high-impact approach has exhibited efficacy in reducing neonatal mortality within the first 24 hours post-birth in other studies.^41^ Although a pooled analysis was not conducted, results from the individual interventions were not statistically significant for a reduction in PDM among neonates and young infants. Antibiotic usage after discharge to prevent PDM was also commonly studied, likely because this approach targets infection-related mortality, a leading contributor to PDM.^42^ However, the findings did not reveal evidence to establish that antibiotic usage meaningfully reduces PDM. Vitamin A supplementation was also studied, possibly because vitamin A deficiency is common in the region and confers a greater risk of severe illness among children.^43^^,^^44^ However, there was no consistent statistically significant effect supporting the use of micronutrient supplementation interventions to reduce PDM. Although not included in the present study’s analyses, a previous systematic review and meta-analysis of malaria chemoprophylaxis revealed a 77% reduction in mortality compared with placebo among children recovering from severe anemia.^17^

Of the 18 interventions targeting PDM, only two yielded statistically significant reductions in PDM. These included vitamin A supplementation for children admitted with pneumonia in Tanzania^31^ and a pilot study conducted to assess the impact of an access to services intervention for children admitted with sickle cell disease in Tanzania.^33^ Notably, other identified studies involving similar interventions within these categories did not reveal statistical significance, potentially indicating context-dependent variability in outcomes in PDM prevention. Furthermore, many of the included publications had low-quality evidence per GRADE. Thus, additional high-quality studies assessing interventions to prevent PDM are warranted.

The interventions identified in the present systematic review predominantly targeted specific populations of children defined by admission diagnoses (e.g., pneumonia, malnutrition, anemia, etc.) and were tailored to underlying disease processes (e.g., supplemental feeding after discharge for children with malnutrition and KMC for neonates with low birth weight). Although two identified interventions included components such as caregiver education and linkage to follow-up care, most interventions inadequately addressed the broader social determinants of PDM, including caregiver health, economic constraints, and barriers to healthcare access, that contribute to PDM risk.

This gap highlights the need for holistic care models that integrate biomedical, health system, and psychosocial interventions to prevent mortality across all discharged populations, irrespective of initial diagnosis.^45^ The heterogeneity of study populations, along with small sample sizes, likely contributed to the inconsistent effectiveness observed across studies. The authors of future studies should prioritize adequately powered PDM trials to evaluate comprehensive post-discharge care packages tailored to high-risk children identified using validated clinical risk assessment tools, thereby optimizing resource allocation and the impact of interventions.

### Limitations.

It is important to consider the limitations of the present systematic review. Despite a comprehensive search strategy across multiple databases, the study approach may have resulted in missing studies on interventions known or hypothesized to reduce PDM that were not captured by the specific keywords used. Additionally, a gray literature review was not conducted because the field of PDM reduction is still relatively new, which may limit the inclusion of PDM reduction work in policy or programming documents. When evaluating system-based interventions, such as linkage-to-care programs, individual randomized controlled trials may not be ideal. Rather, cluster-randomized controlled trials may provide more appropriate evidence by accounting for implementation and delivery challenges in a region and not at the individual level. Given the use of published results, age-specific analyses could not be conducted in the present study. This is of particular importance because, although some interventions to prevent PDM targeted neonatal populations (e.g., KMC), others did not differentiate outcomes among neonates. Mortality can be a rare outcome, and therefore, individual studies may not have been sufficiently powered to detect reductions in PDM. The authors of future reviews may expand to include studies examining outcomes such as nutritional status, health-seeking behaviors, or other clinical markers of improvement that may lead to mortality reduction.

## CONCLUSION

The current body of research on interventions for preventing PDM among children in sub-Saharan Africa has involved supplemental feeding programs, KMC, antibiotic use, and micronutrient supplementation. Their effectiveness in preventing PDM among children varied considerably both within and between intervention types, with only a few revealing statistically significant reductions in PDM. This heterogeneity in efficacy underscores the need for further research to identify and implement cost-effective, practical interventions that can reliably reduce PDM among children in sub-Saharan Africa. Such additional studies would facilitate meta-analyses that ultimately determine the efficacy of individual interventions.

## Supplemental Materials

10.4269/ajtmh.25-0567 Supplemental Materials
