# Supplementary material for: Interventions to Prevent Post-Discharge Mortality among Children in Sub-Saharan Africa: A Systematic Review
Source: Am J Trop Med Hyg. 2026 Feb 17;114(4):787–95. doi: 10.4269/ajtmh.25-0567 (PMC13045548; doi:10.4269/ajtmh.25-0567)
Supplement: Supplemental Materials [file tpmd250567.SD1.pdf]

Appendix. Search terms used to identify publications reporting on risk assessment tools for post-discharge mortality among children in sub-Saharan Africa

PubMed 1857 07/31/2024

(“post-discharge mortal*”[tw] OR “post discharge mortal*”[tw] OR PDM) OR (("Hospitalization"[Mesh] OR hospital*[tw]) AND ("Mortality"[Mesh] OR mortal*[tw] OR death*[tw] OR fatal*[tw]) AND ("Follow-Up Studies"[Mesh] OR "Longitudinal Studies"[Mesh] OR followup[tw] or “follow up”[tw] OR “follow-up”[tw] OR postdischarge[tw] OR post-discharge[tw] OR “post discharge”[tw] OR “after discharge”[tw] OR “after-discharge”[tw] OR “after hospital*”[tw] OR “after-hospital”[tw] OR “post hospital*”[tw] OR “post-hospital*”[tw] OR “patient discharge”[tw]))

("Adolescent"[Mesh] OR adolesc*[tw] OR baby[tw] OR babies[tw] OR boy[tw] OR boys[tw] OR child*[tw] OR "Child"[Mesh] OR girl*[tw] OR Infan*[tw] OR "Infant"[Mesh] OR juvenil*[tw] OR minors[tw] OR neonat*[tw] OR neo-nat*[tw] OR newborn*[tw] OR new-born*[tw] OR pediat*[tw] OR paediat*[tw] OR "Pediatrics"[Mesh] OR "Pediatricians"[Mesh] OR perinat*[tw] OR pre-adolescen*[tw] OR preadolescen*[tw] OR prematur*[tw] OR prepubesc*[tw] OR pre-pubesc*[tw] OR pubescen*[tw] OR "school age*"[tw] OR schoolchild*[tw] OR "school child*"[tw] OR teen*[tw] OR toddler*[tw] OR under*age*[tw] OR young[tw] OR "young adult"[MeSH Terms] OR youth*[tw] OR preschool*[tw] OR "pre-school"[tw] OR kindergarden*[tw] OR kindergarten*[tw] OR "elementary school*"[tw] OR "middle school*"[tw] OR "junior high*"[tw] OR "primary school*"[tw] OR "secondary school*"[tw] OR "high school*"[tw])

(Angola[tw] OR Benin[tw] OR Botswana[tw] OR "Burkina Faso"[tw] OR Burundi[tw] OR "Cabo Verde"[tw] OR "Cape Verde"[tw] OR Cameroon[tw] OR “Central African Republic”[tw] OR Chad[tw] OR Comoros[tw] OR Congo[tw] OR "Cote d'Ivoire"[tw] OR "Ivory Coast"[tw] OR Djibouti [tw] OR "Equatorial Guinea" [tw] OR Eritrea [tw] OR Eswatini [tw] OR Ethiopia [tw] OR Gabon [tw] OR Gambia [tw] OR Ghana [tw] OR Guinea [tw] OR "Guinea-Bissau" [tw] OR Kenya [tw] OR Lesotho [tw] OR Liberia[tw] OR Madagascar [tw] OR Malawi [tw] OR Mali [tw] OR Mauritania [tw] OR Mauritius [tw] OR Mozambique [tw] OR Namibia [tw] OR Niger [tw] OR Nigeria [tw] OR Rwanda [tw] OR "Sao Tome"[tw] OR Principe [tw] OR Senegal[tw] OR Seychelles [tw] OR "Sierra Leone"[tw] OR Somalia [tw] OR "South Africa"[tw] OR "Sub Saharan"[tw] OR “sub-sahara*”[tw] OR Sudan [tw] OR Tanzania[tw] OR Togo[tw] OR Uganda[tw] OR Zambia[tw] OR Zimbabwe [tw] OR “southern africa*”[tw] OR “central africa*”[tw] OR “eastern africa*”[tw] OR “east africa*”[tw] OR “western africa*”[tw] OR “west africa*”[tw])

(#1 AND #2 AND #3) NOT ("animals"[MeSH Terms] NOT "humans"[MeSH Terms])

Web of Science 1101 07/31/2024

(“post-discharge mortal*” OR “post discharge mortal*” OR PDM) OR (hospital* AND (mortal* OR death* OR fatal*) AND (followup or “follow up” OR “follow-up” OR postdischarge OR post-discharge OR “post discharge” OR “after discharge” OR “after-discharge” OR “after hospital*” OR “after-hospital” OR “post hospital*” OR “post-hospital*” OR “patient discharge”))

(adolesc* OR baby OR babies OR boy OR boys OR child* OR girl* OR Infan* OR juvenil* OR minors OR neonat* OR neo-nat* OR newborn* OR new-born* OR pediat* OR paediat* OR perinat* OR pre-adolescen* OR preadolescen* OR prematur* OR prepubesc* OR pre-pubesc* OR pubescen* OR "school age*" OR schoolchild* OR "school child*" OR teen* OR toddler* OR under*age* OR young OR youth* OR preschool* OR "pre-school" OR kindergarden* OR kindergarten* OR "elementary school*" OR "middle school*" OR "junior high*" OR "primary school*" OR "secondary school*" OR "high school*")
(Angola OR Benin OR Botswana OR "Burkina Faso" OR Burundi OR "Cabo Verde" OR "Cape Verde" OR Cameroon OR “Central African Republic” OR Chad OR Comoros OR Congo OR "Cote d'Ivoire" OR "Ivory Coast" OR Djibouti OR "Equatorial Guinea" OR Eritrea OR Eswatini OR Ethiopia OR Gabon OR Gambia OR Ghana OR Guinea OR "Guinea-Bissau" OR Kenya OR Lesotho OR Liberia OR Madagascar OR Malawi OR Mali OR Mauritania OR Mauritius OR Mozambique OR Namibia OR Niger OR Nigeria OR Rwanda OR "Sao Tome" OR Principe OR Senegal OR Seychelles OR "Sierra Leone" OR Somalia OR "South Africa" OR "Sub Saharan" OR “sub-sahara*” OR Sudan OR Tanzania OR Togo OR Uganda OR Zambia OR Zimbabwe OR “southern africa*” OR “central africa*” OR “eastern africa*” OR “east africa*” OR “western africa*” OR “west africa*”
#1 AND #2 AND #3)

EMBASE 3469 07/31/2024
(“post-discharge mortal*” OR “post discharge mortal*” OR PDM)
(hospital* AND (mortal* OR death* OR fatal*) AND (followup or “follow up” OR “follow-up” OR postdischarge OR post-discharge OR “post discharge” OR “after discharge” OR “after-discharge” OR “after hospital*” OR “after-hospital” OR “post hospital*” OR “post-hospital*” OR “patient discharge”))
(adolesc* OR baby OR babies OR boy OR boys OR child* OR girl* OR Infan* OR juvenil* OR minors OR neonat* OR neo-nat* OR newborn* OR new-born* OR pediat* OR paediat* OR perinat* OR pre-adolescen* OR preadolescen* OR prematur* OR prepubesc* OR pre-pubesc* OR pubescen* OR "school age*" OR schoolchild* OR "school child*" OR teen* OR toddler* OR under*age* OR young OR youth* OR preschool* OR "pre-school" OR kindergarden* OR kindergarten* OR "elementary school*" OR "middle school*" OR "junior high*" OR "primary school*" OR "secondary school*" OR "high school*")
(Angola OR Benin OR Botswana OR "Burkina Faso" OR Burundi OR "Cabo Verde" OR "Cape Verde" OR Cameroon OR “Central African Republic” OR Chad OR Comoros OR Congo OR "Ivory Coast" OR Djibouti OR "Equatorial Guinea" OR Eritrea OR Eswatini OR Ethiopia OR Gabon OR Gambia OR Ghana OR Guinea OR "Guinea-Bissau" OR Kenya OR Lesotho OR Liberia OR Madagascar OR Malawi OR Mali OR Mauritania OR Mauritius OR Mozambique OR Namibia OR Niger OR Nigeria OR Rwanda OR "Sao Tome" OR Principe OR Senegal OR Seychelles OR "Sierra Leone" OR Somalia OR "South Africa" OR "Sub Saharan" OR “sub-sahara*” OR Sudan OR Tanzania OR Togo OR Uganda OR Zambia OR Zimbabwe OR “southern africa*” OR “central africa*” OR “eastern africa*” OR “east africa*” OR “western africa*” OR “west africa*”)
(#1 OR #2) AND #3 AND #4
Limits: humans, embase, article/it, conference paper/it, review/it

Cochrane Reviews 15 Cochrane Trials 507 07/31/2024

(“post-discharge mortal*” OR “post discharge mortal*” OR PDM) OR (hospital* AND (mortal* OR death* OR fatal*) AND (followup or “follow up” OR “follow-up” OR postdischarge OR post-discharge OR “post discharge” OR “after discharge” OR “after-discharge” OR “after hospital*” OR “after-hospital” OR “post hospital*” OR “post-hospital*” OR “patient discharge”))
(adolesc* OR baby OR babies OR boy OR boys OR child* OR girl* OR Infan* OR juvenil* OR minors OR neonat* OR neo-nat* OR newborn* OR new-born* OR pediat* OR paediat* OR perinat* OR pre-adolescen* OR preadolescen* OR prematur* OR prepubesc* OR pre-pubesc* OR pubescen* OR "school age*" OR schoolchild* OR "school child*" OR teen* OR toddler* OR under*age* OR young OR youth* OR preschool* OR "pre-school" OR kindergarden* OR kindergarten* OR "elementary school*" OR "middle school*" OR "junior high*" OR "primary school*" OR "secondary school*" OR "high school*")
(Angola OR Benin OR Botswana OR "Burkina Faso" OR Burundi OR "Cabo Verde" OR "Cape Verde" OR Cameroon OR “Central African Republic” OR Chad OR Comoros OR Congo OR "Cote d'Ivoire" OR "Ivory Coast" OR Djibouti OR "Equatorial Guinea" OR Eritrea OR Eswatini OR Ethiopia OR Gabon OR Gambia OR Ghana OR Guinea OR "Guinea-Bissau" OR Kenya OR Lesotho OR Liberia OR Madagascar OR Malawi OR Mali OR Mauritania OR Mauritius OR Mozambique OR Namibia OR Niger OR Nigeria OR Rwanda OR "Sao Tome" OR Principe OR Senegal OR Seychelles OR "Sierra Leone" OR Somalia OR "South Africa" OR "Sub Saharan" OR “sub-saharan” OR Sudan OR Tanzania OR Togo OR Uganda OR Zambia OR Zimbabwe OR “southern africa” OR “central africa” OR “eastern africa” OR “east africa” OR “western africa” OR “west africa”)
#1 AND #2 AND #3

CAB Global Health 5 07/31/2024
(“post-discharge mortal*” OR “post discharge mortal*” OR PDM) OR (hospital* AND (mortal* OR death* OR fatal*) AND (followup or “follow up” OR “follow-up” OR postdischarge OR post-discharge OR “post discharge” OR “after discharge” OR “after-discharge” OR “after hospital*” OR “after-hospital” OR “post hospital*” OR “post-hospital*” OR “patient discharge”))
(adolesc* OR baby OR babies OR boy OR boys OR child* OR girl* OR Infan* OR juvenil* OR minors OR neonat* OR neo-nat* OR newborn* OR new-born* OR pediat* OR paediat* OR perinat* OR pre-adolescen* OR preadolescen* OR prematur* OR prepubesc* OR pre-pubesc* OR pubescen* OR "school age*" OR schoolchild* OR "school child*" OR teen* OR toddler* OR under*age* OR young OR youth* OR preschool* OR "pre-school" OR kindergarden* OR kindergarten* OR "elementary school*" OR "middle school*" OR "junior high*" OR "primary school*" OR "secondary school*" OR "high school*")
(Angola OR Benin OR Botswana OR "Burkina Faso" OR Burundi OR "Cabo Verde" OR "Cape Verde" OR Cameroon OR “Central African Republic” OR Chad OR Comoros OR Congo OR "Cote d'Ivoire" OR "Ivory Coast" OR Djibouti OR "Equatorial Guinea" OR Eritrea OR Eswatini OR Ethiopia OR Gabon OR Gambia OR Ghana OR Guinea OR "Guinea-Bissau" OR Kenya OR Lesotho OR Liberia OR Madagascar OR Malawi OR Mali OR Mauritania OR Mauritius OR Mozambique OR Namibia OR Niger OR Nigeria OR Rwanda OR "Sao Tome" OR Principe OR Senegal OR Seychelles OR "Sierra Leone" OR Somalia OR "South Africa" OR "Sub Saharan" OR “sub-sahara*” OR Sudan OR Tanzania OR

Togo OR Uganda OR Zambia OR Zimbabwe OR “southern africa*” OR “central africa*” OR “eastern africa*” OR “east africa*” OR “western africa*” OR “west africa*”)
#1 AND #2 AND #3

ProQuest Disserations and Theses 65 07/31/2024
noft((“post-discharge mortal*” OR “post discharge mortal*” OR PDM) OR (hospital* AND (mortal* OR death* OR fatal*) AND (followup or “follow up” OR “follow-up” OR postdischarge OR post-discharge OR “post discharge” OR “after discharge” OR “after-discharge” OR “after hospital*” OR “after-hospital” OR “post hospital*” OR “post-hospital*” OR “patient discharge”))) AND noft(adolesc* OR baby OR babies OR boy OR boys OR child* OR girl* OR Infan* OR juvenil* OR minors OR neonat* OR neo-nat* OR newborn* OR new-born* OR pediat* OR paediat* OR perinat* OR pre-adolescen* OR preadolescen* OR prematur* OR prepubesc* OR pre-pubesc* OR pubescen* OR "school age*" OR schoolchild* OR "school child*" OR teen* OR toddler* OR under*age* OR young OR youth* OR preschool* OR "pre-school" OR kindergarden* OR kindergarten* OR "elementary school*" OR "middle school*" OR "junior high*" OR "primary school*" OR "secondary school*" OR "high school*") AND noft(Angola OR Benin OR Botswana OR "Burkina Faso" OR Burundi OR "Cabo Verde" OR "Cape Verde" OR Cameroon OR “Central African Republic” OR Chad OR Comoros OR Congo OR "Cote d'Ivoire" OR "Ivory Coast" OR Djibouti OR "Equatorial Guinea" OR Eritrea OR Eswatini OR Ethiopia OR Gabon OR Gambia OR Ghana OR Guinea OR "Guinea-Bissau" OR Kenya OR Lesotho OR Liberia OR Madagascar OR Malawi OR Mali OR Mauritania OR Mauritius OR Mozambique OR Namibia OR Niger OR Nigeria OR Rwanda OR "Sao Tome" OR Principe OR Senegal OR Seychelles OR "Sierra Leone" OR Somalia OR "South Africa" OR "Sub Saharan" OR “sub-saharan” OR Sudan OR Tanzania OR Togo OR Uganda OR Zambia OR Zimbabwe OR “southern africa” OR “central africa” OR “eastern africa” OR “east africa” OR “western africa” OR “west africa”)

Supplemental Table 1. Risk of Bias 2 (ROB-2) assessment for included interventions

Author, Year	Randomization Process	Deviations from Intended Intervention	Missing Outcome Data	Measurement of Outcome	Selection of Reported Result	Overall Risk of Bias
Supplemental Feeding						
**Kiguli S, et al. *EClinicalMedicine*. 2024.**	Low risk	Low risk	Low risk	Low risk	Low risk	Low risk
**Rollins NC, et al. *Acta Paediatr.* 2007.**	Low risk	Some concerns	Low risk	Low risk	Some concerns	Some concerns
**Walsh K, et al. *Br J Nutr*. 2024.**	Low risk	Low risk	Low risk	Low risk	Low risk	Low risk
**Kerac M et al. *Lancet.* 2009.**	Low risk	Low risk	Low risk	Low risk	Low risk	Low risk
Kangaroo Mother Care						
**Brotherton H, et al. *EclinicalMedicine*. 2021.**	Some concerns	Low risk	Low risk	Low risk	Low risk	Some concerns
**Kambarami RA, et al. *Ann Trop Paediatr.* 2003.**	*Ineligible for ROB-2 grade as this intervention was not a randomized control trial*					
**Nagai S, et al. *Acta Paediatr*. 2011.**	Low risk	Low risk	Low risk	Low risk	Low risk	Low risk
Antibiotics						
**Berkley JA, et al. *Lancet Glob Health*. 2016.**	Low risk	Low risk	Low risk	Low risk	Low risk	Low risk
**Pavlinac PB, et al. *Lancet Glob Health*. 2021.**	Low risk	Low risk	Low risk	Low risk	Low risk	Low risk
**Maitland K, et al. *Lancet Glob Health*. 2019.**	Low risk	Low risk	Low risk	Low risk	Low risk	Low risk
Micronutrient Supplementation						
**Benn CS, et al. *BMJ*. 2010.**	Low risk	Low risk	Low risk	Low risk	Some concerns	Some concerns
**Fawzi, et al. *Pediatr Infect Dis J*. 1999.**	Low risk	Some concerns	Low risk	Low risk	Some concerns	Some concerns
**Maitland K, et al. *Lancet Glob Health.* 2019.**	Low risk	Low risk	Low risk	Low risk	Low risk	Low risk
**Makonnen B, et al. *J Trop Pediatr*. 2003.**	Low risk	Some concerns	Low risk	Low risk	Some concerns	Some concerns
Other Interventions						
**Aaby et al. *J Infectious Diseases.* 2011. (BCG Vaccination)**	Some concerns	Low risk	Low risk	Low risk	Low risk	Some concerns
**Hau DK, et al. *J Pediatr.* 2021. (Linkage to care)**	*Ineligible for ROB-2 grade as this intervention was not a randomized control trial*					
**Njuguna, et al. *Topics in antiviral medicine.* 2016. (ART).**	Some concerns	Low risk	Low risk	Low risk	Low risk	Some concerns
**Wiens MO, et al. *Glob Health Sci Pract*. 2016. (Referrals + Prevention items)**	*Ineligible for ROB-2 grade as this intervention was not a randomized control trial*					

Supplemental Table 2. Efficacy of interventions to prevent post-discharge mortality

	Experienced Post-Discharge Mortality, n (%)				
**Reference**	**Control Group**	**Intervention Group**	**Hazards/Risk Ratio (95% CI)**	***P*-value**	**Level of Certainty**
Supplemental Feeding					
**Kiguli S, et al. *EClinicalMedicine*. 2024.**	14/422 (3%)	13/424 (3%)	Adjusted HR 1.00 (0.46, 2.15)	Not reported	Low
**Rollins NC, et al. *Acta Paediatr*. 2007.**	18/83 (22%)	25/86 (29%)	Not available	Not reported	Very low
**Walsh K, et al. *Br J Nutr*. 2024.**	12/80 (15%)	11/80 (14%)	HR 0.91 (0.40, 2.07)	0.83	Very low
**Kerac M et al. *Lancet.* 2009.**	67/396 (19%)	47/399 (14%)	RR 0.71 (0.51, 1.00)	0.05	Moderate
Kangaroo Mother Care					
**Brotherton H, et al. *EclinicalMedicine*. 2021.**	34/139 (24%)	29/138 (21%)	RR 0.84 (0.55, 1.29)	0.42	Very low
**Kambarami RA, et al. *Ann Trop Paediatr.* 2003.**	Not reported	79/297 (27%)	Not available	Not reported	Very low
**Nagai S, et al. *Acta Paediatr*. 2011.**	2/36 (6%)	2/36 (6%)	RR 1.00 (0.15, 6.72)	1	Very low
Antibiotics					
**Berkley JA, et al. *Lancet Glob Health*. 2016.**	135/891 (15%)	122/887 (14%)	HR 0.90 (0.71, 1.16)	0.43	Moderate
**Pavlinac PB, et al. *Lancet Glob Health*. 2021.**	19/697 (3%)	15/703 (2%)	HR 0.79 (0.39, 1.58)	0.49	Very low
**Maitland K, et al. *Lancet Glob Health*. 2019.**	163/1989 (8%)	172/1994 (9%)	HR 1.07 (0.86, 1.32)	0.56	Low
Micronutrient Supplementation					
**Benn CS, et al. *BMJ*. 2010.**	78/863 (9%)	83/854 (10%)	Adjusted RR 1.08 (0.79, 1.47)	Not reported	Very low
**Fawzi, et al. *Pediatr Infect Dis J*. 1999.**	34/322 (11%)	18/326 (6%)	HR 0.51 (0.29, 0.90)	0.02	Low
**Maitland K, et al. *Lancet Glob Health.* 2019.**	166/1986 (8%) (iron and folate)	169/1997 (9%) (multivitamin arm)	HR 0.97 (0.79, 1.21)	0.81	Low
**Makonnen B, et al. *J Trop Pediatr*. 2003.**	1/150 (0.9%)	1/150 (0.7%)	Not available	Not reported	Very low
Other Interventions					
**Aaby et al. *J Infectious Diseases.* 2011. (BCG Vaccination)**	124/1161	105/1182	RR 0.83 (0.63, 1.08)	Not reported	Very low
**Hau DK, et al. *J Pediatr.* 2021. (Linkage to care)**	11/57 (19%)	4/59 (7%)	Adjusted HR 0.26 (0.08, 0.83)	0.023	Low
**Njuguna, et al. *Topics in antiviral medicine.* 2016. (Antiretroviral therapy)**	18/93 (20%)	21/90 (23%)	Adjusted HR 1.30 (0.69, 2.45)	0.41	Very low
**Wiens MO, et al. *Glob Health Sci Pract*. 2016. (Referrals + Prevention items)**	41/1242 (3%)	5/202 (3%)	OR 0.75 (0.29, 1.92)	Not reported	Very low

Supplemental Table 3. Grades of Recommendation, Assessment, Development, and Evaluation (GRADE) assessment for level of certainty.

	Study Design	Risk of Bias	Inconsistency	Indirectness	Imprecision	Publication Bias	Overall Level of Certainty
Supplemental Feeding							
**Kiguli S, et al. *EClinicalMedicine*. 2024.**	Open label phase 2 randomized control trial	0	-1	0	0	0	Moderate
**Rollins NC, et al. *Acta Paediatr.* 2007.**	Randomized non-blinded study	-1	-1	0	-2	0	Very low
**Walsh K, et al. *Br J Nutr*. 2024.**	Open label phase II trial	0	-1	0	-2	0	Very low
**Kerac M et al. *Lancet.* 2009.**	Double blind randomized placebo-controlled efficacy trial	0	-1	0	0	0	Moderate
Kangaroo Mother Care							
**Brotherton H, et al. *EclinicalMedicine*. 2021.**	Nonblinded pragmatic RCT	-1	-1	0	-1	0	Very low
**Kambarami RA, et al. *Ann Trop Paediatr.* 2003.**	Prospective descriptive study	0	-1	0	-2	0	Very low
**Nagai S, et al. *Acta Paediatr*. 2011.**	Randomized control trial with long term follow up	0	-1	0	-2	0	Very low
Antibiotics							
**Berkley JA, et al. *Lancet Glob Health*. 2016.**	Multicentre, double-blind, randomized, placebo-controlled study	0	-1	0	0	0	Moderate
**Pavlinac PB, et al. *Lancet Glob Health*. 2021.**	Double blind placebo controlled RCT	0	-1	0	-2	0	Very low
**Maitland K, et al. *Lancet Glob Health*. 2019.**	Two stratum-open label multicentre factorial randomized tract trial	0	-1	0	-1	0	Low
Micronutrient Supplementation							
**Benn CS, et al. *BMJ*. 2010.**	Randomized placebo controlled two by two factorial trial	0	-1	0	0	0	Moderate
**Fawzi, et al. *Pediatr Infect Dis J.* 1999.**	Randomized double blind placebo, controlled trial	-1	-1	0	0	0	Low
**Maitland K, et al. *Lancet Glob Health.* 2019.**	Two stratum-open label multicenter factorial randomized tract trial	0	-1	0	-1	0	Low
**Makonnen B, et al. *J Trop Pediatr*. 2003.**	Randomized controlled trial	0	-1	0	-2	0	Very low
Other Interventions							
**Aaby et al. *J Infectious Diseases.* 2011. (BCG Vaccination)**	Randomized placebo controlled two by two factorial trial	-1	-1	0	-1	0	Very low
**Hau DK, et al. *J Pediatr.* 2021. (Linkage to care)**	Pilot study	0	-1	0	0	0	Very low (not a randomized controlled trial)
**Njuguna, et al. *Topics in antiviral medicine.* 2016. (ART)**	Unmasked randomized controlled trial	0	-1	0	-2	0	Very low
**Wiens MO, et al. *Glob Health Sci Pract*. 2016. (Referrals + Prevention items)**	Proof of concept study	0	-1	0	-2	0	Very low
